# Supplementary material for: Identification and Evaluation of Sugarcane Cultivars for Antixenosis Resistance to the Leafhopper Yamatotettix flavovittatus Matsumura (Hemiptera: Cicadellidae)
Source: Plants (Basel). 2024 Aug 18;13(16):2299. doi: 10.3390/plants13162299 (PMC11360375; doi:10.3390/plants13162299)
Supplement: Supplementary file 1 [file plants-13-02299-s001.zip › plants-3038990-supplementary.pdf]

Supplements

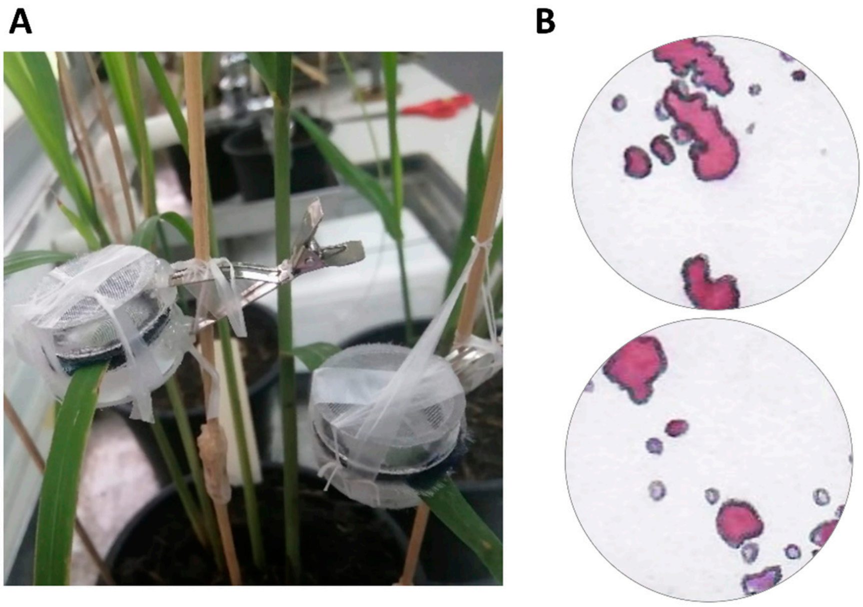

Supplemental Figure S1.

(A) Collection of leafhopper *Y. flavovittatus* honeydew on Whatman filter paper that was placed at the bottom of insect cages, and (B) representative staining of honeydew spots on filter paper with 0.1% ninhydrin.

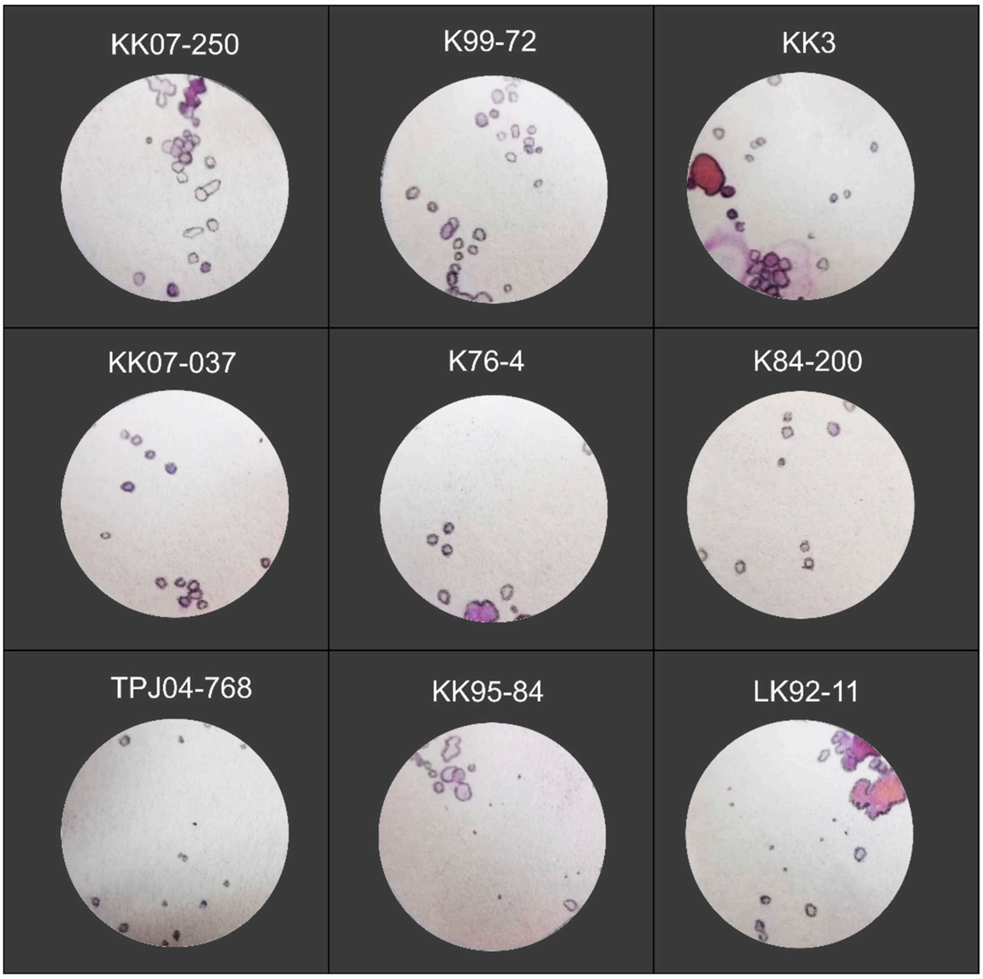

Supplemental Figure S2.

Honeydew excretion on filter paper. The size of the honeydew area and the intensity of the honeydew color correspond to the leafhopper *Y. flavovittatus* feeding activity.

Supplemental Table S1. List of parameters used for cluster analysis and their significance levels from test statistics using Mann–Whitney U-test and Tukey–Kramer (SAS software).

No.	Parameters	Significance level
1	Average settling behavior of leafhopper 24 h [Settling 24 h]	< 0.001
2	Average settling behavior of leafhopper 48 h [Settling 48 h]	< 0.001
3	Average settling behavior of leafhopper 72 h [Settling 72 h]	< 0.001
4	Average settling behavior of leafhopper 96 h [Settling 96 h]	< 0.001
5	Average honeydew production area 5 h [Honeydew 5 h]	< 0.001
6	Average honeydew production area 10 h [Honeydew 10 h]	< 0.001
7	Percentage of waveform Np duration [WDEI NP]	0.002
8	Percentage of waveform Yf1 duration [WDEI Yf1]	0.415
9	Percentage of waveform Yf2 duration [WDEI Yf2]	0.045
10	Percentage of waveform Yf3 duration [WDEI Yf3]	0.016
11	Percentage of waveform Yf4 duration [WDEI Yf4]	0.034
12	Percentage of waveform Yf5 duration [WDEI Yf5]	0.203
13	Percentage of waveform Np frequency [NEWI NP]	0.003
14	Percentage of waveform Yf1 frequency [NEWI Yf1]	0.020
15	Percentage of waveform Yf2 frequency [NEWI Yf2]	0.031
16	Percentage of waveform Yf3 frequency [NEWI Yf3]	0.161
17	Percentage of waveform Yf4 frequency [NEWI Yf4]	0.009
18	Percentage of waveform Yf5 frequency [NEWI Yf5]	0.705
19	No. Silica cells per 100 µm [No. Si cells]	< 0.001
20	No. Silica row per 100 µm [No. Si rows]	< 0.001
21	Trichome density per 100 µm [Trichome density]	< 0.001
